# Impact of Early Life Famine Exposure on Body Composition and Metabolic Profiles in Adulthood

**DOI:** 10.1111/mcn.13777

**Published:** 2024-12-17

**Authors:** Shuaihua Song, Liyuan Zhang, Hanze Du, Yuelun Zhang, Yue Jiang, Daowei Li, Yi Hu, Shi Chen, Huijuan Zhu, Guangliang Shan, Hui Pan

**Affiliations:** ^1^ Key Laboratory of Endocrinology of National Health Commission, Department of Endocrinology, State Key Laboratory of Complex Severe and Rare Diseases Peking Union Medical College Hospital, Chinese Academy of Medical Science & Peking Union Medical College Beijing China; ^2^ Center for Prevention and Early Intervention, National Infrastructures for Translational Medicine, Institute of Clinical Medicine Peking Union Medical College Hospital, Chinese Academy of Medical Science and Peking Union Medical College Beijing China; ^3^ Jilin Provincial Key Laboratory of Tooth Development and Bone Remodeling School and Hospital of Stomatology, Jilin University Changchun China; ^4^ State Key Laboratory of Complex, Severe, and Rare Diseases, Biomedical Engineering Facility of National Infrastructures for Translational Medicine Peking Union Medical College Hospital Beijing China; ^5^ Department of Epidemiology and Statistics Institute of Basic Medical Sciences, Chinese Academy of Medical Sciences & School of Basic Medicine, Peking Union Medical College Beijing China

**Keywords:** adulthood, body composition, famine, malnutrition, metabolic profiles

## Abstract

The relationship between the famine and metabolic syndrome has been reported, but there is a lack of more detailed changes in metabolic profiles. It is unclear how famine affects body composition. This study included 21,142 participants from the China National Health Survey. The body mass index (BMI), fat mass index (FMI), and fat‐free mass index (FFMI) were calculated. Systolic blood pressure (SBP), diastolic blood pressure (DBP), blood lipids, and fasting blood glucose (FBG) were measured. Multivariate adjusted linear regression models were used to assess the association between famine and outcome. Our results shown that fetal‐exposed group had higher BMI and FMI (*β* > 0). Childhood‐exposed group showed an average decrease of 0.08 standard deviation (SD) in FFMI, and adolescence‐exposed group had lower BMI and FFMI than non‐exposed group. SBP were 0.38 SD higher in fetal‐exposed group, 0.58 SD higher in childhood‐exposed group and 0.85 SD higher in adolescence‐exposed group than non‐exposed group. Famine‐exposed groups had higher total cholesterol (TC), low density lipoprotein‐cholesterol (LDL‐C), and FBG levels (*β* > 0). For females with famine exposure, they had a higher BMI, FMI, LDL‐C, TG, and TC than males. Overall, early famine exposure is associated with increased blood pressure, LDL‐C, TC, and FBG. Muscle mass loss in adulthood associated with childhood and adolescence famine exposure. Famine‐exposed females appear to have higher levels of body fat and blood lipids.

## Summary

- Early‐life famine exposure is linked to increased FMI, blood pressure, total cholesterol (TC), LDL‐C, and fasting blood glucose (FBG) levels, with notable effects observed in fetal‐exposed and childhood‐exposed groups.
- Childhood and adolescence famine exposure is associated with decreased muscle mass in adulthood, indicating potential long‐term effects on body composition.
- Females exposed to famine show higher levels of body fat, LDL‐C, triglycerides (TG), and TC compared to males, suggesting a gender‐specific vulnerability to metabolic changes following early‐life famine.
- Rural‐born individuals exposed to famine during childhood and adolescence had lower BMI and FMI, but higher lipid levels, suggesting that famine may have had a significant impact on lipid metabolism.

## Graphical abstract

Early life famine exposure was closely related to sarcopenia, hypertension, hyperglycemia and dyslipidemia in later life, and the impact of famine exposure on females seems to be more significant than males. This study might provide evidence of long‐term famine effects on body composition.

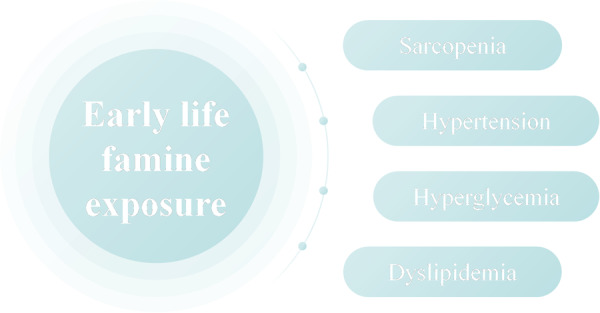

## 1 Introduction

In recent years, body composition has gained increasing attention. Lean body mass and fat mass are associated with the development and progression of many chronic metabolic diseases, such as obesity and type 2 diabetes, and body composition has also been reported to be associated with increased all‐cause mortality (Eglseer et al. 2023, Bell et al. 2022, de Ritter et al. 2023, Chang et al. 2023, Yu et al. 2022). Body mass index (BMI) is a traditional and commonly index to assess human body weight and nutritional status, but BMI are unable to present information on muscle and adipose tissue (Ying et al. 2023). Individuals with the same BMI have large differences in their body composition, and fat mass index (FMI) and fat‐free mass index (FFMI) may better reflect the health status of individuals (Ying et al. 2023, Sedlmeier et al. 2021, Sørensen, Frederiksen, and Heitmann 2020). Body composition is highly heritable, but it is also influenced by many factors, such as diet, exercise (Livingstone et al. 2021). For example, chronic malnutrition can lead to decreased muscle mass in individuals, while overeating can lead to subcutaneous or visceral fat accumulation (Fielding et al. 2023, de Lauzon‐Guillain et al. 2017). Moreover, BMI, FMI, and FFMI are closely associated with blood pressure, serum lipids and fasting blood glucose (FBG) which are common metabolic profiles. Hypertension, diabetes and dyslipidemia have caused a huge burden on society and medical care system. It is necessary to prevent metabolic disorder.

The developmental origins of health and disease (DOHaD) theory suggests that early life, including fetal life, childhood, and adolescence, is a critical period affecting the development of growth, metabolism, and nervous system (Hoffman, Reynolds, and Hardy 2017). As early as 1986, Professor Barker found that intrauterine fetal dysplasia was strongly associated with an increased risk of cardiovascular disease in adulthood, and a review found the same results (Barker 1986, Victora et al. 2008). Maternal diet and nutritional status during pregnancy play a critical role in fetal growth and offspring health (Hoffman et al. 2021). Data from a study in the United States showed that poor maternal diet was associated with increased neonatal obesity (Shapiro et al. 2017). A Nepalese study showed that maternal supplementation with folic acid, iron, and zinc during pregnancy contributed to the children with lower fat mass (Stewart et al. 2009). In addition, early life development has also been shown to be closely related to the development of type 2 diabetes in adulthood, especially in individuals with low birth weight who have a significantly increased risk of type 2 diabetes in adulthood (Wang et al. 2022, Whincup et al. 2008).

The Great Famine in China from 1959 to 1962 was considered the severe famine of the 20th century, with more than 30 million deaths (Mu and Zhang 2011). Numerous factors contributed to this nationwide famine, including the Great Leap Forward which was an economic and social campaign and droughts, etc. (Smil 1999). The national famine has caused most people being in a state of severe inadequate energy intake for a long time, which provides a unique opportunity to observe long‐term outcomes of chronic malnutrition, and helps us understand the association between malnutrition in early life and metabolic status in adulthood (Zhang et al. 2022). Current research on individual metabolic status and famine mostly focuses on the risk of metabolic syndrome, the long‐term effects of famine on body composition are not clear. Therefore, we explored the effects of early famine exposure on body composition and metabolic profiles, based on data from China National Health Survey (CNHS).

## 2 Methods

### 2.1 Data Source and Study Population

This is an observational study and data was analyzed from the CNHS (9 provinces‐Gansu, Guizhou, Hainan, Heilongjiang, Inner Mongolia, Qinghai, Shanxi, Xinjiang, and Yunnan). CNHS is a national cross‐sectional survey conducted since 2012, which uses a multistage stratified cluster sampling method to select a representative population from each province of Chinese Mainland. The specific protocol of this survey has been published (He et al. 2018), and this investigation is still ongoing. All interviewers and staff were trained to ensure that they were able to complete face‐to‐face interviews correctly and smoothly.

**Figure 1:**
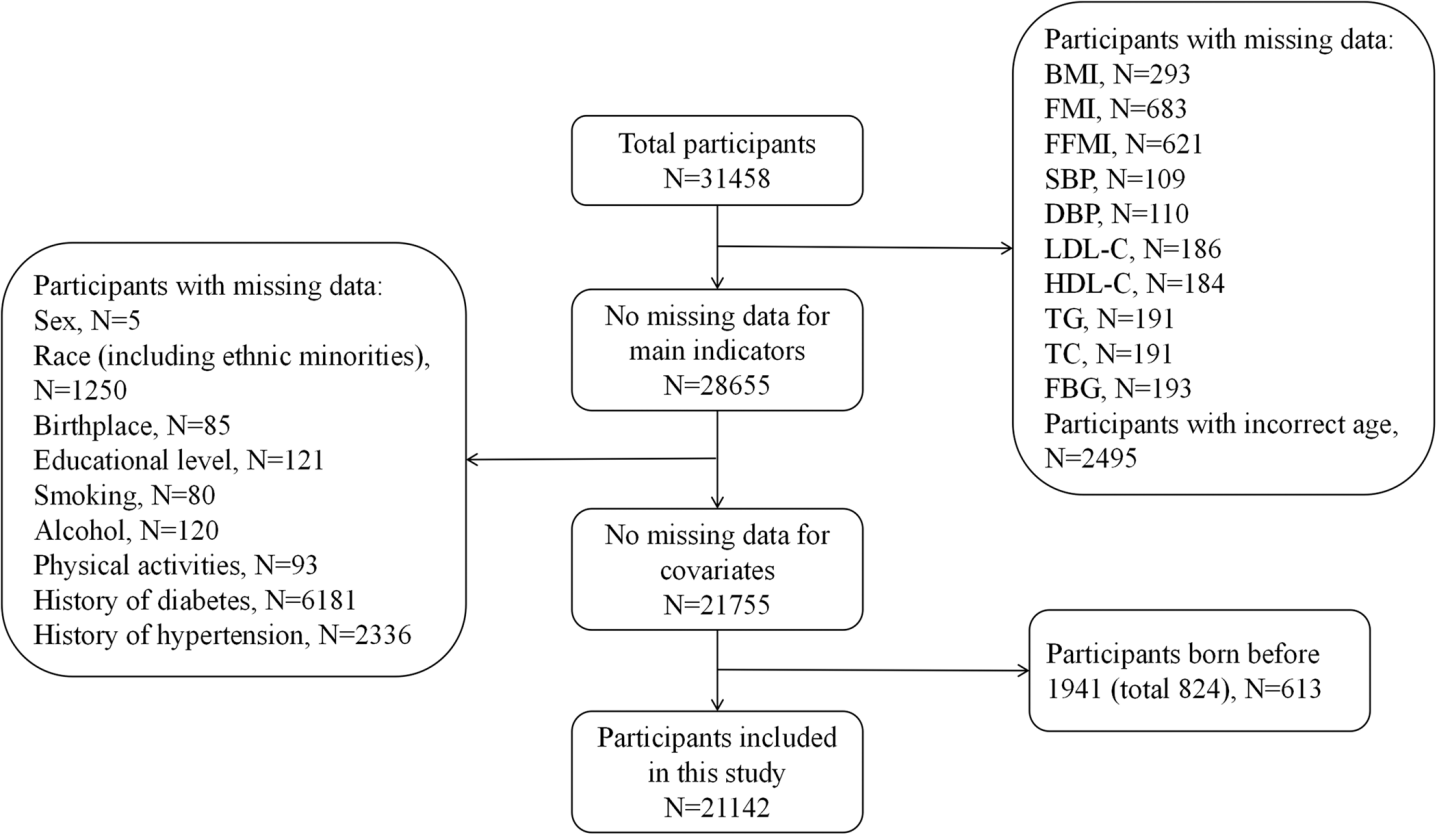
Summary of participants flow diagram.

Considering genetic heterogeneity, we only included Han people over 20 years. Participants with incomplete data, including sex, race, birthplace, educational level, smoking, alcoho1, physical labor, history of diabetes, history of hypertension, body composition, systolic blood pressure (SBP), diastolic blood pressure (DBP), blood lipids and fasting blood glucose, were also excluded in this study. In addition, our study focused on the effects of early life famine exposure, so we excluded participants born before January 1, 1941 (famine exposure in adulthood). A total of 21,142 participants included in the final analysis. The details were shown in Figure 1.

### 2.2 Definition of Famine Exposure Status

In the current study, fetal exposure was defined as the date of birth from 1/1/1959 to 31/12/1962, childhood exposure was defined as the date of birth from 1/1/1949 to 31/12/1958, adolescent exposure was defined as the date of birth from 1/1/1941 to 31/12/1948, and non‐famine exposure was defined as the date of birth after 1/1/1963 (Zhang et al. 2022, Lu et al. 2020). Childhood was defined as age 0–10 years and adolescence was defined as age 10–18 years.

### 2.3 Clinical Laboratory Tests and Anthropometric Measurements

Participants wore light clothing and were barefoot for anthropometric measurements. Height was measured to the nearest 0.1 cm using a fixed stadiometer. Body weight, fat mass was measured by a body composition analyzer (TANITA BC‐420, Japan) with a precision of 1 decimal place. BMI was calculated as weight in kilograms divided by height in square meters (kg/m^2^). FMI was calculated as fat mass index in kilograms divided by height in square meters (kg/m^2^). FFMI was calculated as fat‐free mass in kilograms divided by height in square meters (kg/m^2^) (Baker et al. 2022).

Blood pressure (BP) measurements were performed in the morning by trained personnel (mmHg). During the measurement, the right upper limb of participants was fully exposed, and each individual was measured three times using a digital blood pressure measurement device (Omron HEM‐907, Japan), recording the mean values of SBP and DBP, respectively. Before BP measurements, all participants were asked to rest for at least 5 min and to refrain from exercise, alcohol, smoking, or tea for at least 30 min.

All participants were asked to fast for at least 8 h the night before the examination and a fasting blood sample was taken by professional nurses in the next morning. Separated plasma or serum was frozen in aliquots and kept at −80°C until first thawed for analysis. Serum lipids (mmol/L), including total cholesterol (TC), triglyceride (TG), low density lipoprotein‐cholesterol (LDL‐C), high density lipoprotein‐cholesterol (HDL‐C), and FBG levels, were measured using a chemistry analyzer (Cholesterol Cobas8000C701, USA).

### 2.4 Covariates

Demographic information was recorded using a standardized questionnaire, including birthday, sex, place of birth (urban or rural areas), and education level (less than high school, high school or equivalent, college or above). Lifestyle collection was also performed by standardized questionnaires including smoking (never, former, and current), alcohol consumption (never, former, and current), physical labor (light, moderate, and heavy). Never smoker was defined as smoking never in life. Former smoker was defined as smoking in life but no smoking in the last 6 months, and current smokers were those who reported smoking cigarettes “every day” or “some days.” Never drinker was defined as never drinking in life. Former drinker was defined as having drank in life but not in the last 6 months, and current drinker were those who reported drinking “every day” or “some days.” Physical labor was defined according to self‐reported occupation. Light physical labor consists mainly of sedentary work or little walking, such as office work or cashiering. Moderate labor includes tasks that require more movement but are not overly strenuous, like teaching or delivery driving, while heavy labor involves high physical demand, such as construction work or farm labor. Data on history of diabetes mellitus and hypertension was from self‐reports (yes or no). The specific criteria for defining hyperlipidemia include: TC ≥ 6.2 mmol/L, LDL‐C ≥ 4.1 mmol/L, TG ≥ 2.3 mmol/L, and/or HDL‐C < 1.0 mmol/L (Li et al. 2023).

### 2.5 Statistical Analyses

For continuous variables, data was presented as median (p25, p75) due to non‐normal distribution, and the analysis of Kruskal–Wallis test was performed. The Bonferroni method post‐hoc tests were made in Kruskal–Wallis test, and *p* value was performed for multiple comparisons by the Bonferroni method. For categorical variables, data was presented as number (percentage), and Pearson *χ* ^2^ was performed.

Multivariate adjusted linear regression models were used in this study to assess the association of famine exposure with outcome variables and regression coefficients and their 95% confidence intervals were calculated. These models were adjusted for age, sex, place of birth, education level, smoking, alcohol consumption, and physical labor. We also performed stratified analyses according to covariates and assessed their multiplicative interaction with famine exposure. To minimize the impact of age on this study, we used restriction approach as a sensitivity analysis.

Only the Bonferroni method post‐hoc tests were performed using SPSS version 25 and other analyses were performed using Stata version 16. A *p* < 0.05 (two‐tailed) was considered statistical significance.

### 2.6 Ethics Statement

This study was approved by the Institutional Review Board of Institute of Basic Medical Sciences, Chinese Academy of Medical Sciences (No.: 028‐2013).

## 3 Results

### 3.1 Baseline Characteristics

A total of 21,142 participants were included in this study, including 1988 (9.4%) famine exposed during fetal life, 4615 (21.8%) famine exposed during childhood, and 1783 (8.4%) famine exposed during adolescence (Table 1). The BMI (23.2 [21.0, 25.7]), FMI (6.3 [4.9, 7.9]), SBP (116 [107, 126]), DBP (74 [67, 81]), LDL‐C (2.6 [2.1, 3.1]), TG (1.3 [0.9, 1.9]), TC (4.5 [3.9, 5.1]), and FBP (4.9 [4.6, 5.3]) in non‐famine exposed group were significantly lower than early life famine exposed groups and there were significant statistical differences between non‐exposed group and the famine‐exposed groups (*p* < 0.001). In addition, multiple comparisons between groups showed that LDL‐C and TC levels were not significantly different among the three famine‐exposed subgroups, but were significantly different compared with non‐exposed group (*p* < 0.05). There were significant differences in SBP and FBG levels between fetal‐exposed group and childhood‐exposed group (*p* < 0.05), and there were also significant differences in BMI, FMI, SBP, DBP, TG, and FBG levels between fetal‐exposed group and adolescent‐exposed group (*p* < 0.05). In addition, BMI, SBP, and DBP levels differed between the childhood‐exposed and adolescent‐exposed groups (*p* < 0.05).

**Table 1:** Baseline characteristics of participants from China National Health Survey.

	Non‐exposed group	Fetal‐exposed group	Childhood‐exposed group	Adolescence‐exposed group
Characteristics	*N* = 12,756 (60.3)	*N* = 1988 (9.4)	*N* = 4615 (21.8)	*N* = 1783 (8.4)
Age, years*	41.0 (33.0, 46.0)	53.0 (52.0, 54.0)	60.0 (57.0, 62.0)	69.0 (67.0, 71.0)
Gender*				
Male	4905 (59.3)	753 (9.1)	1863 (22.5)	748 (9.1)
Female	7851 (61.0)	1235 (9.6)	2752 (21.4)	1035 (8.0)
Birthplace*				
Urban areas	4542 (61.0)	844 (11.3)	1588 (21.3)	475 (6.4)
Rural areas	8214 (60.0)	1144 (8.4)	3027 (22.1)	1308 (9.6)
Education*				
Less than high school	4593 (45.9)	852 (8.5)	3213 (32.1)	1356 (13.5)
High school or equivalent	2789 (59.0)	731 (15.5)	923 (19.5)	288 (6.1)
College or above	5374 (84.0)	405 (6.3)	479 (7.5)	139 (2.2)
Smoking*				
Never	9394 (61.5)	1415 (9.3)	3194 (20.9)	1260 (8.3)
Former	474 (34.9)	126 (9.3)	520 (38.3)	238 (17.5)
Current	2888 (63.9)	447 (9.9)	901 (19.9)	285 (6.3)
Alcohol*				
Never	8241 (58.2)	1359 (9.6)	3226 (22.8)	1335 (9.4)
Former	494 (40.4)	155 (12.7)	397 (32.5)	176 (14.4)
Current	4021 (69.8)	474 (8.2)	992 (17.2)	272 (4.7)
Physical labor*				
Light	9301 (59.2)	1436 (9.1)	3491 (22.2)	1479 (9.4)
Moderate	1716 (71.7)	197 (8.2)	377 (15.8)	102 (4.3)
Heavy	1739 (57.2)	355 (11.7)	747 (24.5)	202 (6.6)
Diabetes*				
No	12423 (62.5)	1810 (9.1)	4114 (20.7)	1535 (7.7)
Yes	333 (26.4)	178 (14.1)	501 (39.8)	248 (19.7)
Hypertension*				
No	11513 (66.3)	1510 (8.7)	3225 (18.6)	1108 (6.4)
Yes	1243 (32.8)	478 (12.6)	1390 (36.7)	675 (17.8)
Hyperlipidemia*				
No	9121 (64.3)	1198 (8.4)	2765 (19.5)	1098 (7.7)
Yes	3635 (52.2)	790 (11.4)	1850 (26.6)	685 (9.8)
BMI, kg/m^2^*	23.2 (21.0, 25.7)^a,b,c^	24.3 (22.2, 26.6)^a,d^	24.2 (22.1, 26.5)^b,e^	24.0 (21.7, 26.4)^c,d,e^
FMI, kg/m^2^*	6.3 (4.9, 7.9)^a,b,c^	7.1 (5.6, 8.8)^a,d^	7.0 (5.3, 9.0)^b^	6.9 (4.9, 9.1)^c,d^
FFMI, kg/m^2^*	16.4 (15.4, 18.4)^a,b^	16.6 (15.7, 18.6)^a^	16.7 (15.7, 18.5)^b^	16.6 (15.7, 18.1)
SBP, mmHg*	116 (107, 126)^a,b,c^	125 (114, 137)^a,d,e^	129 (118, 142)^b,d,f^	134 (122, 149)^c,e,f^
DBP, mmHg*	74 (67, 81)^a,b,c^	78 (72, 87)^a,d^	78 (71, 86)^b,e^	77 (69, 84)^c,d,e^
LDL‐C, mmol/L*	2.6 (2.1, 3.1)^a,b,c^	3.0 (2.4, 3.6)^a^	3.0 (2.5, 3.6)^b^	3.0 (2.5, 3.7)^c^
HDL‐C, mmol/L	1.3 (1.1, 1.6)	1.3 (1.1, 1.6)	1.3 (1.1, 1.6)	1.3 (1.1, 1.6)
TG, mmol/L*	1.3 (0.9, 1.9)^a,b,c^	1.6 (1.1, 2.3)^a,d^	1.5 (1.1, 2.3)^b^	1.5 (1.1, 2.1)^c,d^
TC, mmol/L*	4.5 (3.9, 5.1)^a,b,c^	5.0 (4.4, 5.7)^a^	5.0 (4.4, 5.7)^b^	5.1 (4.4, 5.8)^c^
FBG, mmol/L*	4.9 (4.6, 5.3)^a,b,c^	5.2 (4.8, 5.7)^a,d,e^	5.3 (4.9, 5.8)^b,d^	5.3 (4.9, 6.0)^c,e^

*Note:* Data are *n* (%) and median (p25, p75). *There were statistical differences among different famine exposed groups (*p* < 0.001). ^a,b,c^The same superscript indicates statistically significant difference between the two groups (*p* < 0.05).

Abbreviations: BMI, body mass index; DBP, diastolic blood pressure; FBG, fasting blood glucose; FFMI, fat‐free mass index; FMI, fat mass index; HDL‐C, high‐density lipoprotein cholesterol; LDL‐C, low‐density lipoprotein cholesterol; SBP, systolic blood pressure; TC, total cholesterol; TG, triglyceride.

### 3.2 Correlation Analysis

In this study, the results of the multivariate adjusted linear regression model indicated that fetal‐exposed had higher BMI (*β* [95% CI] = 0.13 [0.08–0.17]) and FMI (*β* [95% CI] = 0.16 [0.12–0.20]) compared with non‐exposed group (Table 2). The results of childhood famine exposure and FMI were similar. Compared with the reference group, childhood‐exposed group showed an average decrease of 0.08 standard deviation (SD) in FFMI, and adolescence‐exposed group showed an average decrease of 0.13 SD in BMI and 0.21 SD in FFMI.

**Table 2:** Association between different famine exposed group and body composition/metabolic profiles.

	Non‐exposed group	Fetal‐exposed group		Childhood‐exposed group		Adolescence‐exposed group	
Variables*	*β* (95% CI)	*β* (95% CI)	*p*‐values	*β* (95% CI)	*p*‐values	*β* (95% CI)	*p*‐values
BMI, kg/m^2^	Ref.	0.13 (0.08 to 0.17)	< 0.001	0.01 (−0.03 to 0.04)	0.660	−0.13 (−0.18 to −0.08)	< 0.001
FMI, kg/m^2^	Ref.	0.16 (0.12 to 0.20)	< 0.001	0.07 (0.04 to 0.10)	< 0.001	−0.01 (−0.05 to 0.04)	0.884
FFMI, kg/m^2^	Ref.	0.01 (−0.02 to 0.04)	0.409	−0.08 (−0.10 to −0.06)	< 0.001	−0.21 (−0.24 to −0.18)	< 0.001
SBP, mmHg	Ref.	0.38 (0.33 to 0.42)	< 0.001	0.58 (0.55 to 0.61)	< 0.001	0.85 (0.80 to 0.89)	< 0.001
DBP, mmHg	Ref.	0.27 (0.23 to 0.32)	< 0.001	0.21 (0.18 to 0.25)	< 0.001	0.07 (0.02 to 0.12)	0.003
LDL‐C, mmol/L	Ref.	0.39 (0.34 to 0.43)	< 0.001	0.46 (0.42 to 0.49)	< 0.001	0.55 (0.50 to 0.60)	< 0.001
HDL‐C, mmol/L	Ref.	0.07 (0.03 to 0.12)	0.001	0.11 (0.07 to 0.14)	< 0.001	0.15 (0.11 to 0.20)	< 0.001
TG, mmol/L	Ref.	0.15 (0.11 to 0.20)	< 0.001	0.07 (0.03 to 0.10)	< 0.001	−0.02 (−0.07 to 0.03)	0.370
TC, mmol/L	Ref.	0.42 (0.37 to 0.47)	< 0.001	0.46 (0.42 to 0.50)	< 0.001	0.53 (0.48 to 0.58)	< 0.001
FBG, mmol/L	Ref.	0.28 (0.23 to 0.32)	< 0.001	0.32 (0.28 to 0.35)	< 0.001	0.38 (0.33 to 0.43)	< 0.001

*Note:* All models were adjusted for sex, birthplace, educational level, smoking, drinking and physical labor. Models of BMI, FMI, FFMI were additionally adjusted the history of diabetes, hypertension and hyperlipidemia. Models of LDL‐C, HDL‐C, TG, TC were additionally adjusted BMI, the history of diabetes, hypertension. Models of SBP and DBP were additionally adjusted BMI, the history of diabetes and hyperlipidemia. Model of FBG was additionally adjusted BMI, the history of hypertension and hyperlipidemia. *All outcome variables were standardized (per SD).

Abbreviations: BMI, body mass index; DBP, diastolic blood pressure; FBG, fasting blood glucose; FFMI, fat‐free mass index; FMI, fat mass index; HDL‐C, high‐density lipoprotein cholesterol; LDL‐C, low‐density lipoprotein cholesterol; SBP, systolic blood pressure; TC, total cholesterol; TG, triglyceride.

Participants with famine exposure had higher SBP and DBP. The results showed that SBP were 0.38 SD higher in fetal‐exposed group, 0.58 SD higher in childhood‐exposed group and 0.85 SD higher in adolescence‐exposed group than the non‐famine exposed group. Our results also showed that fetal famine exposure has the higher impact on DBP (*β* [95% CI] = 0.27 [0.23–0.32]), while adolescence famine exposure has the lower impact (*β* [95% CI] = 0.07 [0.02–0.12]).

Participants with famine exposure also had higher LDL‐C, HDL‐C, TC, and FBG levels (*β* > 0). And the levels of these indicators showed an upward trend from the fetal‐exposed group to the adolescent‐exposed group. The TG level of individuals with fetal (*β* [95% CI] = 0.15 [0.11–0.20]) or childhood famine exposure (*β* [95% CI] = 0.07 [0.03–0.10]) was higher than individuals without famine exposure. However, there was no association between adolescent famine exposure and TG level.

We repeated the linear regression analysis after excluding participants under 45 years old (Table 3). There was no significant correlation between fetal famine exposure and BMI/FMI/HDL‐C. Individuals with childhood famine exposure had a lower BMI, FMI than reference (*β* [95% CI] = −0.11 [−0.15 to −0.07], −0.04 [−0.08 to −0.01], respectively). Similarly, participants in adolescence‐exposed group had a lower FMI and DBP than non‐exposed group (*β* [95% CI] = −0.12 [−0.17 to −0.07], −0.08 [–0.13 to −0.02], respectively). There was a negative association between adolescent famine exposure and TG level (*β* [95% CI] = −0.11 [−0.17 to −0.05]). Other results were generally consistent with the main analysis.

**Table 3:** Association between famine exposure and body composition/metabolic profiles, excluded participants under 45 years.

	Non‐exposed group	Fetal‐exposed group		Childhood‐exposed group		Adolescence‐exposed group	
Variables*	*β* (95% CI)	*β* (95% CI)	*p*‐values	*β* (95% CI)	*p*‐values	*β* (95% CI)	*p*‐values
BMI, kg/m^2^	Ref.	−0.01 (−0.05 to 0.04)	0.873	−0.11 (−0.15 to −0.07)	< 0.001	−0.25 (−0.30 to −0.20)	< 0.001
FMI, kg/m^2^	Ref.	0.03 (−0.08 to −0.01)	0.194	−0.04 (−0.08 to −0.01)	0.018	−0.12 (−0.17 to −0.07)	< 0.001
FFMI, kg/m^2^	Ref.	−0.04 (−0.07 to −0.01)	< 0.001	−0.13 (−0.16 to −0.11)	< 0.001	−0.27 (−0.30 to −0.23)	< 0.001
SBP, mmHg	Ref.	0.21 (0.16 to 0.26)	< 0.001	0.42 (0.38 to 0.46)	< 0.001	0.69 (0.63 to 0.74)	< 0.001
DBP, mmHg	Ref.	0.11 (0.06 to 0.17)	< 0.001	0.06 (0.02 to 0.10)	0.005	−0.08 (−0.13 to −0.02)	0.006
LDL‐C, mmol/L	Ref.	0.23 (0.17 to 0.28)	< 0.001	0.31 (0.26 to 0.35)	< 0.001	0.40 (0.34 to 0.45)	< 0.001
HDL‐C, mmol/L	Ref.	0.04 (−0.01 to 0.09)	0.161	0.07 (0.03 to 0.11)	< 0.001	0.12 (0.06 to 0.17)	< 0.001
TG, mmol/L	Ref.	0.06 (0.01 to 0.11)	0.034	−0.07 (−0.09 to 0.02)	0.284	−0.11 (−0.17 to −0.05)	< 0.001
TC, mmol/L	Ref.	0.22 (0.17 to 0.28)	< 0.001	0.28 (0.24 to 0.32)	< 0.001	0.35 (0.29 to 0.40)	< 0.001
FBG, mmol/L	Ref.	0.13 (0.07 to 0.19)	< 0.001	0.17 (0.12 to 0.22)	< 0.001	0.23 (0.16 to 0.29)	< 0.001

*Note:* All models were adjusted for sex, birthplace, educational level, smoking, drinking and physical labor. Models of BMI, FMI, FFMI were additionally adjusted the history of diabetes, hypertension and hyperlipidemia. Models of LDL‐C, HDL‐C, TG, TC were additionally adjusted BMI, the history of diabetes, hypertension. Models of SBP and DBP were additionally adjusted BMI, the history of diabetes and hyperlipidemia. Model of FBG was additionally adjusted BMI, the history of hypertension and hyperlipidemia. *All outcome variables were standardized (per SD).

Abbreviations: BMI, body mass index; DBP, diastolic blood pressure; FBG, fasting blood glucose; FFMI, fat‐free mass index; FMI, fat mass index; HDL‐C, high‐density lipoprotein cholesterol; LDL‐C, low‐density lipoprotein cholesterol; SBP, systolic blood pressure; TC, total cholesterol; TG, triglyceride.

### 3.3 Stratified Analysis

**Figure 2:**
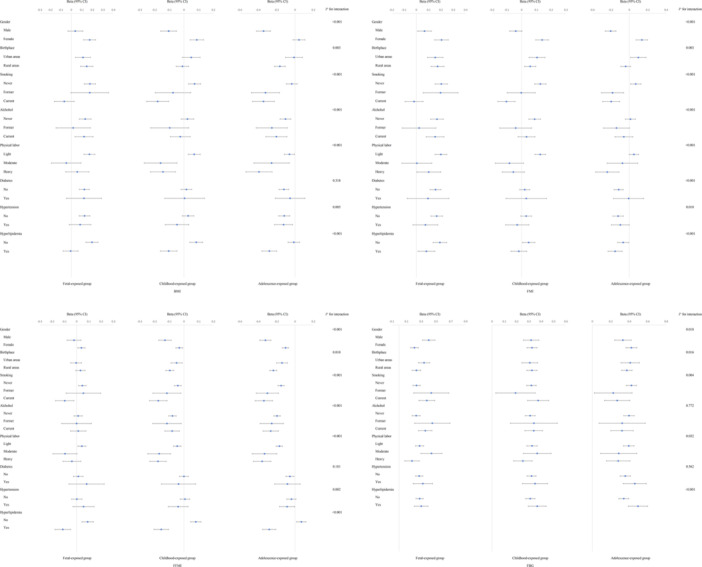
The stratification analysis of BMI, FMI, FFMI, and FBG. BMI, body mass index; FBG, fasting blood glucose; FFMI, fat‐free mass index; FMI, fat mass index.

We further explored the association between famine exposure and outcomes in different subgroups by performing stratified analyses according to sex, place of birth, smoking, alcohol consumption, physical labor, history of hypertension, diabetes and Hyperlipidemia. The results stratified by sex showed that participants with childhood had lower BMI and FMI than reference in males, but higher in females (Figure 2). For females with childhood and adolescence famine exposure, they had a higher FFMI than males. Rural‐born participants exposed to famine during childhood and adolescence had lower FFMI compared to urban‐born participants. Higher BMI and FMI levels among rural‐born exposed to famine during fetal life and lower levels during childhood and adolescence. Additionally, in the subgroup with diabetes and hypertension, no association was found between famine exposure and BMI/FMI/FFMI. Our results suggested that there were interactions between famine exposure and gender, place of birth, smoking, drinking, physical labor history of hypertension and hyperlipidemia for BMI (excluding the adolescent exposure group)/FMI/FFMI.

**Figure 3:**
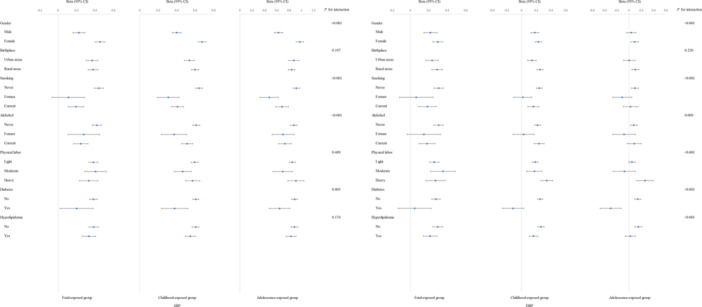
The stratification analysis of SBP and DBP. DBP, diastolic blood pressure; SBP, systolic blood pressure.

**Figure 4:**
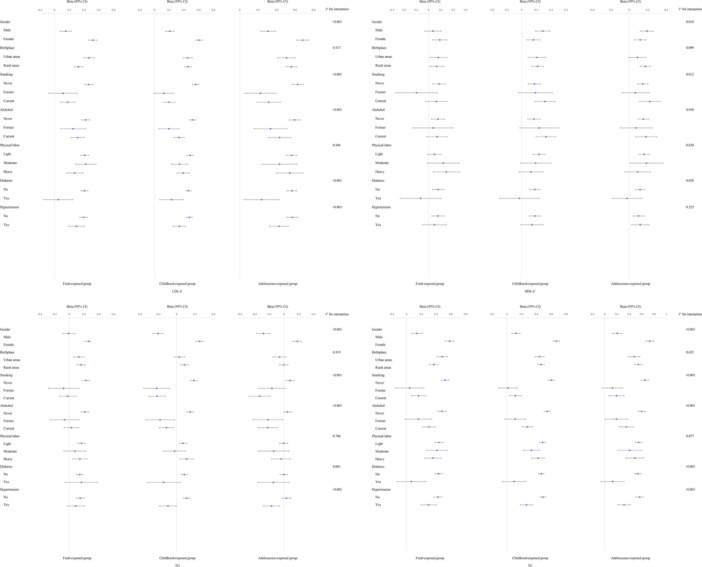
The stratification analysis of blood lipid. HDL‐C, high‐density lipoprotein cholesterol; LDL‐C, low‐density lipoprotein cholesterol; TC, total cholesterol; TG, triglyceride.

Generally, the results of the stratified analyses of blood pressure were consistent with the linear regression. SBP tended to increase in all subgroups, while DBP tended to decrease (Figure 3). Similarly, the stratified analyses results of LDL‐C, HDL‐C, TC, and FBG levels were generally consistent with linear regression (Figures 2 and 4). Lower TG levels in males with childhood and adolescent famine exposure, while females had higher TG levels. Higher SBP and DBP levels in rural‐born individuals exposed to famine in fetal and childhood, and higher lipid levels in rural‐born individuals exposed to famine in childhood and adolescence. However, the FBG level appear to be higher in urban‐born individuals exposed to famine during fetal life and adolescence. The results of the stratified analysis were shown in Figures 2 and 4.

## 4 Discussion

The major global famines experienced in the past include the Leningrad Famine (1941–1944), the Dutch Famine (Winter of 1944–1945), the Chinese Famine (1959–1961), the Nigerian Famine (1967–1970), the Ethiopian Famine (1983–1985), and the Brazilian Famine (2021–2022). The Chinese Great Famine was considered to be more serious and health‐hazardous.

This research included 21,142 Han people in 9 provinces across distinct geographic and economic regions in China, including the northwest, southwest, northeast, central, and southern areas. It also included key agricultural such as Heilongjiang, reflecting the regional diversity during the period of the Great Famine. These regional differences provide broader representativeness for the study. Famine exposure was defined based on birth dates, corresponding to the period of the Chinese Great Famine (1959–1962). This period was characterized by widespread food shortages and malnutrition, as consistently documented in historical records and previous literature. Although regions such as Xinjiang had relatively higher grain production, mortality rates remained high (Meng, Qian, and Yared 2015, Lin and Yang 2000).

Our study analyzed the relationship between body composition and famine exposure, and indicated that famine exposure in early life was linked to increased body fat, blood pressure, blood lipids and FBG, as well as decreased muscle mass in adulthood. Notably, famine exposure in early life might have a more significant effect on females. The research revealed that early life famine exposure has a significant impact on FFMI and FMI in adulthood. High fat mass is usually associated with a series of adverse health outcomes, including type 2 diabetes, hypertension, stroke, impaired physical function, and increased mortality (Woo, Leung, and Kwok 2012). Lean mass is a commonly used indicator reflecting skeletal muscle mass, and low lean mass is also a major component of sarcopenia, which is related to lower physical function levels (Earnest et al. 2010). Our previous research established the normal reference ranges for FFMI and FMI in China, finding gender differences in FFMI and FMI. Females had higher FMI and lower FFMI (Jin et al. 2019). Our stratified analysis indicated that females with famine exposure had higher BMI and FMI in later life. Moreover, there was evidence indicating that “the aging process begins in the womb,” with fetal reactions to malnutrition leading to permanent structural, physiological, and metabolic adaptations, consequently increasing the risk of chronic degenerative diseases in later life, including reduced lean body mass, diminished muscle strength, higher prevalence of age‐related diseases, and reduced cognitive abilities (Bleker et al. 2016, Hirani et al. 2015, Qi et al. 2023). An imbalance of FMI and FFMI during the aging process has been observed to be associated with an increased risk of metabolic heart diseases (He et al. 2023).

Furthermore, this study indicated that the blood pressure, blood lipids, and FBG levels remarkably increased in the famine exposure group. The Dutch famine research indicated that fetal‐famine exposure can lead to an increased SBP and the risk of hypertension at age 59 (Stein et al. 2006). According to the Nigerian famine study, prenatal famine exposure was linked to higher blood pressure and an increased risk of hypertension at age 40 (Miranda et al. 2010). A study from Leningrad indicated that exposure to famine in early life can lead to higher DBP at the age 52 and an increased risk of hypertension at the age 70 (Rotar et al. 2015). Study had shown that prenatal famine exposure can result in intrauterine protein deficiency, leading to endothelial dysfunction and subsequently increased blood pressure (Sinclair et al. 2007). And the “scar hypothesis” suggested that the long‐term negative psychological impact in early life may lead to higher blood pressure (Preston, Hill, and Drevenstedt 1998). Animal studies have also verified that severe early‐life malnutrition can affect hepatic glucose and lipid metabolism (Shen et al. 2019, Lie et al. 2014). It has been reported that malnutrition during adolescence can directly impact the secretion of stress hormones like thyroid hormones, thereby affecting glycolipid metabolism (Jang et al. 2018).

Our stratified analysis showed that females may be more susceptible to early famine, especially in lipid metabolism. Some studies shown that females exposed to famine in early life had a notably higher risk of metabolic disorders than males (Zhang et al. 2022, Qi et al. 2023, Hu et al. 2022, Shao et al. 2021, Liu et al. 2021, Meng et al. 2019). Some scholars proposed that due to the traditional Chinese culture, in tough conditions, parents are more inclined to allocate resources to boys, which may be one of the reasons why males are less affected by famine than females (Mu and Zhang 2011). Interestingly, rural‐born individuals exposed to famine during childhood and adolescence had lower BMI and FMI, but higher lipid levels, suggesting that famine may have had a significant impact on lipid metabolism. Some subgroup results may not correspond with linear regression results. These findings suggested that famine exposure may have complex interactions with smoking and physical labor, however, further research is needed to explore the specific mechanisms involved.

Due to the close relationship between age and date of birth, we did not include age in the regression models. We excluded individuals under the age of 45 and conducted a sensitivity analysis to minimize the age difference between the famine‐exposed and non‐exposed groups. The sensitivity analysis results for the BMI and FMI models showed some differences from the linear regression results, indicating that age might have a substantial impact on BMI and FMI. However, the overall decrease trend of coefficient in our sensitivity analyses is consistent with linear regression, which still suggested famine exposure have an adverse impact on BMI and FMI. However, the potential impact of age in this study should not be ignored. The increasing age is associated with increasing risks of sarcopenia, hypertension, hyperglycemia and dyslipidemia.

There were some limitations in our study. Due to the varying duration and severity of famine in different regions, our exposure subgroups cannot be more precise. This study was a cross‐sectional study and cannot explain causality. Furthermore, restrictions still cannot completely eliminate the impact of age on outcomes, and more caution was needed in explaining the results. Due to lack of data, this study did not consider the effect of dietary factors and medication history on outcomes. Moreover, due to the limited geographical scope of the dataset, the generalizability of these findings to other regions of China not included in this analysis should be approached with caution.

The strengths of our study included a large sample size and a representative population covering multiple regions, which ensured sufficient power in statistical analysis. Additionally, we conducted stratified analysis and provided more detailed information. Importantly, we analyzed the association between famine exposure and body composition, and to reconfirm the gender differences in famine exposure among the Chinese population.

## 5 Conclusion

Early‐life famine exposure is linked to adverse changes in body composition and metabolic profiles. Fetal exposure was related to increased BMI, FMI, blood pressure, LDL‐C, and fasting glucose. Childhood exposure was associated with higher FMI and blood pressure but lower FFMI. Adolescence exposure showed reduced BMI and FFMI, with increased blood pressure and LDL‐C. Additionally, famine‐exposed females appear to have higher levels of body fat and blood lipids. These findings suggest famine exposure elevates long‐term metabolic risks.

## Author Contributions

S.S., L.Z., and H.D. developed the study concept, analysed and interpreted the data. S.S. and L.Z. wrote the manuscript. G.S. and H.P. conducted data curation. Y.Z., Y.J., D.L., Y.H., and S.C. contributed to the discussion and reviewed/edited the manuscript. H.Z., G.S., and H.P. supervised and reviewed/edited the manuscript. H.D., S.C., and G.S. provided financial supporting.

## Conflicts of Interest

The authors declare no conflicts of interest.

## Data Availability

Data will be made available on request.
